# Editor Bids Adieu

**DOI:** 10.4103/0974-2077.79175

**Published:** 2011

**Authors:** Venkataram Mysore

**Affiliations:** *Venkat Charmalaya - Centre for Advanced Dermatology, Bangalore, Karnataka, India*

It is with a sense of happiness and pride that I complete my term as the Editor-in-Chief of the Journal of Cutaneous and Aesthetic Surgery, after a fruitful three years term. It has been a satisfying term as the journal which started as a biannual journal has progressed in to quarterly journal. The number of articles being submitted to the journal website is steadily increasing, and impact factor is also on the rise. Articles (almost 25% of all submissions) are being submitted from across the globe, not only from dermatology, but also from other fields such as plastic surgery, maxillofacial surgery, general surgery etc. We also started focus issues, on several topics such as fillers, hair restoration, sclerotherapy, and the current one on vitiligo surgery. The financial health of the journal is sound, and we managed to publish the issues without taxing the association funds. And most importantly, the journal has got indexed, the first journal in the field of aesthetic and cutaneous surgery in India to do so. That this could be done with in first two years of starting the journal is a matter of great satisfaction.

This could not have happened without the support of several sources- the executive committee (2008-2010) of Association of Cutaneous Surgeons Association (I), headed by Dr. Somesh Gupta, Past presidents Drs Niti Khunger, Narendra Patwardhan, Sharad Mutalik, Secretary Manas Chatterjee have all been very supportive. Our publisher, Dr. Sahu and his diligent staff at Medknow publishers have been truly extraordinary in publishing the journals on time. My sincere thanks to them.

The editorial board members, both national and international, have been very cooperative. I specially single out with gratitude, Dr. Uwe Wollina who was very encouraging at all times. My most sincere thanks are due to my editorial team of assistant and deputy editors, Drs KC Nischal, Kumaresan, Anitha Raghunath, Nandini AS, Anitha BS and HM Omprakash - with out their support, the journal publication would have been impossible.

Lastly, I thank the contributors who submitted excellent articles to the journal and the members of our association who encouraged and appreciated our efforts.

Personally, it has been a truly learning, enjoyable experience, which I will cherish for a long time. I leave with a sense of achievement, as I hand over the baton to Dr. Somesh Gupta, a most capable and dynamic person, under whom, I have no doubt that the journal will scale greater heights.

